# A Digital Patient-Provider Communication Intervention (InvolveMe): Qualitative Study on the Implementation Preparation Based on Identified Facilitators and Barriers

**DOI:** 10.2196/22399

**Published:** 2021-04-08

**Authors:** Berit Seljelid, Cecilie Varsi, Lise Solberg Nes, Kristin Astrid Øystese, Elin Børøsund

**Affiliations:** 1 Department of Digital Health Research Division of Medicine Oslo University Hospital Oslo Norway; 2 Institute of Clinical Medicine Faculty of Medicine University of Oslo Oslo Norway; 3 Department of Cooperation, Patient Education and Equivalent Health Services CEO's Staff Oslo University Hospital Oslo Norway; 4 Faculty of Health and Social Sciences University of South-Eastern Norway Drammen Norway; 5 Department of Psychiatry & Psychology College of Medicine & Science Mayo Clinic Rochester, MN United States; 6 Section of Specialized Endocrinology Department of Endocrinology, Morbid Obesity and Preventive Medicine Oslo University Hospital Oslo Norway; 7 Department of Medical Biochemistry Institute of Clinical Medicine Faculty of Medicine, University of Oslo Oslo Norway

**Keywords:** eHealth, digital communication, secure messages, digital symptom assessment, implementation, tailoring, Consolidated Framework for Implementation Research, CFIR, facilitators, barriers, stakeholders

## Abstract

**Background:**

Chronic health conditions are affecting an increasing number of individuals, who experience various symptoms that decrease their quality of life. Digital communication interventions that enable patients to report their symptoms have been shown to positively impact chronic disease management by improving access to care, patient-provider communication, clinical outcomes, and health-related quality of life. These interventions have the potential to prepare patients and health care providers (HCPs) before visits and improve patient-provider communication. Despite the recent rapid development and increasing number of digital communication interventions that have shown positive research results, barriers to realizing the benefits offered through these types of interventions still exist.

**Objective:**

The aim of this study is to prepare for the implementation of a digital patient-provider communication intervention in the daily workflow at 2 outpatient clinics by identifying potential determinants of implementation using the Consolidated Framework for Implementation Research (CFIR) to tailor the use of digital communication intervention to the intended context and identify key aspects for an implementation plan.

**Methods:**

A combination of focus groups, workshops, and project steering committee meetings was conducted with HCPs (n=14) and patients (n=2) from 2 outpatient clinics at a university hospital. The CFIR was used to guide data collection and analysis. Transcripts, written minutes, and notes were analyzed and coded into 5 CFIR domains using thematic analysis.

**Results:**

Data were examined and analyzed into 18 CFIR constructs relevant to the study purpose. On the basis of the identified determinants, important intervention tailoring includes adjustments to the digital features and adjustments to fit the clinical workflow and a decision to conduct a future pilot study. Furthermore, it was decided to provide the intervention to patients as early as possible in their disease trajectory, with tailored information about its use. Key aspects for the implementation plan encompassed maintaining the identified engagement and positive attitude, involving key stakeholders in the implementation process, and providing the needed support and training.

**Conclusions:**

This study offers insight into the involvement of stakeholders in the tailoring and implementation planning of a digital communication intervention in clinical practice. Stakeholder involvement in the identification of implementation facilitators and barriers can contribute to the tailoring of digital communication interventions and how they are used and can also inform systematic and targeted implementation planning.

## Introduction

### Background

Living with a chronic health condition causes symptoms that negatively affect health-related quality of life (HRQoL) [1-4]. Symptom recognition may be challenging for patients and, by extension, for health care providers (HCPs) [5,6]. This can relate to patients’ poor understanding of disease mechanisms and progression, lack of knowledge, and low levels of health literacy [5,7] and to practical barriers to inform HCPs about symptoms [6]. Experiences of difficulties in communication and interaction between patients and HCPs are common, including poor timing of information and challenging symptom recognition, which are factors that may interfere with symptom management and help seeking [5,7]. eHealth communication interventions may offer the potential to alleviate such difficulties.

Studies of eHealth communication interventions have reported benefits in terms of patient-provider communication [8-14], patient-provider relationship [15], patient self-management [16], symptom management [11,17,18], preparation before hospital visits [9,10,18,19], and HRQoL [8,17,20] in chronic health care settings. Despite these benefits, barriers to benefit realization still exist [21], including staff familiarity with technology [22,23], level of patient education [20,23], and issues with user-friendliness [22,24].

Although the positive effects of eHealth interventions on patient-provider communication and patient outcomes are known, HCPs report concerns regarding the integration of eHealth interventions into daily workflow [25,26] and concerns about increased workload [27-29]. In addition, the use of eHealth interventions can challenge HCPs’ competence [25,26]. Such challenges may act as barriers to implementation and actual use, which could be another barrier for use. Successful implementation of such eHealth interventions requires attention to the development and evaluation of strategies to implement the interventions [30-32]. An important factor for the acceptance and success of eHealth implementation is the tailoring of the interventions to suit the local context [33]. Stakeholders representing the target group and the actual context can provide important input to reduce system complexity, increase system acceptability, and make systems as user-friendly as possible [33,34]. To increase the likelihood of implementation success, there is also a need to examine intervention characteristics from the end-user perspective in order to inform the tailoring of the intervention to suit contextual needs.

Implementation refers to the systematic uptake of research into HCP practice to improve the quality of health care services [35]. Implementation strategies can be explained as methods or techniques used to enhance the adoption, implementation, and sustainability of HCP clinical practice [36]. However, there is limited guidance regarding the types of implementation strategies that may be effective when implementing eHealth interventions to practice [30]. Nevertheless, it has been suggested that implementation strategies should be selected and tailored to address the unique contextual needs based on an identification of determinants (ie, factors that act as facilitators or barriers) that may influence the implementation process [32]. The identification of determinants can be used to address barriers and leverage facilitators [32,37].

Implementation frameworks can guide the identification of determinants that might influence the implementation, its effectiveness, and the implementation process [32,35]. The Consolidated Framework for Implementation Research (CFIR) is a widely used framework to identify facilitators and barriers [38-40]. The CFIR was developed from a synthesis of 20 existing theories and frameworks and consists of 5 overarching domains, including 39 specific constructs within these 5 domains [38]. The first domain of the CFIR is the *Intervention Characteristics* and includes constructs such as the adaptability of the intervention, the perceived relative advantage, and the complexity and cost of the intervention [38]. The *Outer Setting* domain includes constructs such as the patient’s needs and resources related to the intervention, whereas the *Inner Setting* domain includes constructs such as implementation climate and readiness for implementation, the organization’s culture, and leadership engagement. The fourth domain is the *Characteristics of Individuals* involved in the intervention or implementation process; it relates to personal attributes, including personal traits such as motivation, values, and competence. The last domain relates to the *Process* and includes planning, execution, and evaluation of the implementation process [38].

### Objectives

The aim of this study is to prepare the implementation of a digital patient-provider communication intervention, *InvolveMe*, into the daily workflow at 2 outpatient clinics where patients with chronic health conditions are treated by identifying potential facilitators and barriers to implementation using CFIR as the conceptual framework to (1) tailor the *InvolveMe* intervention to the intended context and (2) identify key aspects for an implementation plan.

## Methods

### Overview

**Figure 1:**
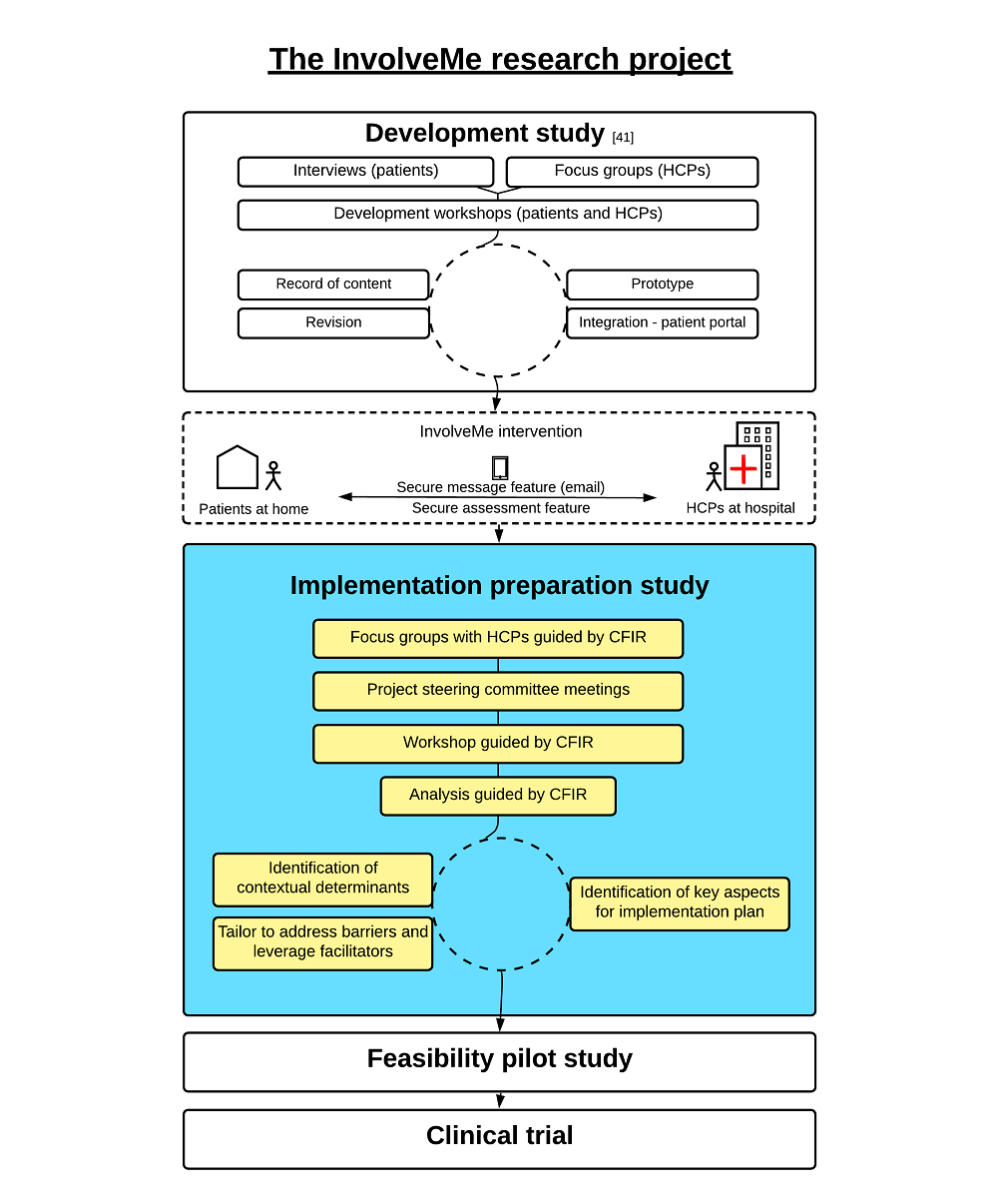
Overview of the InvolveMe research project. CFIR: Consolidated Framework for Implementation Research; HCP: health care providers.

This study is part of the *InvolveMe* research project, which includes the development, implementation, and evaluation of a digital intervention (Figure 1). The *InvolveMe* research project is a collaboration between 2 outpatient clinics and 1 research department at a large university hospital in Norway. The *InvolveMe* intervention will be implemented in 2 outpatient clinics and tested in a future clinical trial (Figure 1).

### Description of the InvolveMe Intervention

**Figure 2:**
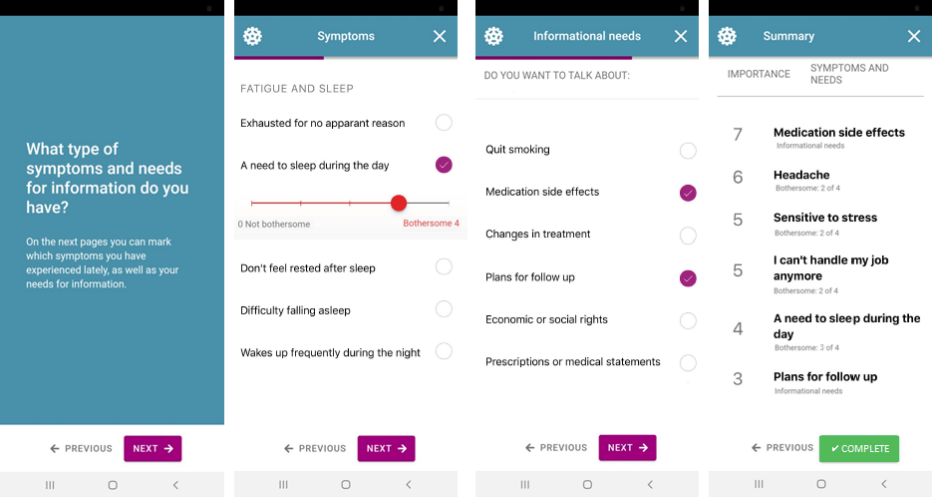
Screenshots of InvolveMe.

*InvolveMe* was developed at the initiative of HCPs aiming to improve follow-up for two specific categories of patients: renal transplant recipients and patients with nonfunctioning pituitary adenomas [41]. *InvolveMe* was internally developed at the hospital in close cooperation with registered nurses, physicians, health support personnel, patients, researchers, and system developers (Figure 1). A detailed description of the content and system development of *InvolveMe* is provided elsewhere [41]. The *InvolveMe* intervention contains two features: (1) a secure message feature and (2) a secure assessment feature (ie, predefined list) where patients can prioritize their need for symptom management, information, and preferences for care from home and on a scale from 0 to 10 (Figure 2).

Patients and HCPs can use the secure message feature to interact with each other between or after outpatient visits [41]. Completion of the secure assessment feature generates a summary that is sent to the patients’ HCPs for use in upcoming consultations. The secure message(s) and the assessment(s) are integrated into an existing patient portal that allows patients to read their electronic patient record (EPR). However, the opposite is not possible (ie, the EPR cannot receive data from the patient portal) owing to information safety regulations.

### Design

This study used a participatory and iterative design approach [42] using qualitative methods for data collection. The data collection period was November 2017 to December 2018 and proceeded through focus groups, project steering committee meetings, and a workshop (Figure 1). Data collection from focus groups and workshops was guided by CFIR [38] (Multimedia Appendices 1 and 2). The variety of data collection activities and diverse data collection approaches allowed for mutual stakeholder learning and comprehension of all stakeholder perspectives involved [43]. As part of the main focus of this study is to identify local determinants to tailor the intervention into the intended context (ie, HCPs’ practice) and identify key aspects for an implementation plan, most study participants represented the HCP perspective. However, to ensure that the patient perspective was also continuously involved, 2 patients with experience from the *InvolveMe* development study [41] were included in the steering committees to ensure knowledge, inclusion, and prioritization of the patient perspective(s).

The determinants identified from data collection and analysis informed the tailoring of the *InvolveMe* intervention to suit workflow at the 2 participating outpatient clinics and aided in the identification of key aspects to include in an implementation plan. Tailoring, as described in this study, refers to addressing intervention barriers and leveraging facilitators as key aspects of the implementation planning process.

### Data Collection Guided by CFIR

CFIR allows researchers to select constructs that they perceive as most relevant and use them to guide the assessment of determinants in the implementation context [38]. The operationalization of CFIR domains in this study was based on discussion and consensus in the research team, where all 5 domains of CFIR were explored for the development of focus group and workshop guides (Multimedia Appendices 1 and 2). Significant themes were to be discussed for determinants important for tailoring the intervention and providing input for the implementation plan (ie, key aspects). By asking about themes to discuss within each domain, rather than questions for each construct, several CFIR constructs would most likely not be covered by the focus group and workshop guides.

#### Focus Groups

The focus group guide centered around HCPs’ experiences from previous successful implementation projects, their perception of possible advantages and challenges of using a digital patient-provider communication intervention, and how to successfully implement the *InvolveMe* intervention (Multimedia Appendix 1).

#### Workshop

The workshop guide consisted of the 5 operationalized domains of CFIR (Multimedia Appendix 2): (1) the *InvolveMe* intervention (*Intervention Characteristics*); (2) the patients who will be offered the *InvolveMe* intervention (*Outer Setting*); (3) the 2 outpatient clinics where the patients are being treated (*Inner Setting*); (4) the HCPs (*Characteristics of Individuals*); and (5) the preparation for implementation of the *InvolveMe* intervention (*Process*).

### Settings, Participants, and Recruitment

Participants were HCPs and patients. HCPs were purposely selected and recruited from an outpatient nephrology clinic and an outpatient endocrine clinic at a large university hospital in Norway. They were registered nurses, physicians, and health support personnel responsible for the treatment and care of patients with renal transplants or nonfunctioning pituitary adenomas. HCPs were provided with written information about the study and those willing to participate were included. The patients were participants in the development study [41] (Figure 1). They were asked to participate in the project steering committees, representing each of the 2 categories of patients. This was based on detailed knowledge about the *InvolveMe* research project [41], in addition to their own experience of being a patient. The patients were contacted by HCPs at the clinics and asked to participate before being contacted by the first author (BS), who described study participation in detail before the final study participation agreement was received.

HCPs had 2 to 38 years of clinical experience from specialist health care, with a median of 49.5 years (range 28-63 years), and most were female (10/14, 71%). Some HCPs participated in all data collection activities, whereas others participated in 1 or 2 activities (Table 1). The patient participants were female.

**Table 1:** Overview of participants in the focus groups, project steering committees, and workshop.

Stakeholders^a^			Focus groups (n=11)		Project steering committee (n=6)		Workshop (n=7)
**Endocrine outpatient clinic**							
	Head of clinic (physician)			✓^b^		✓	
	Registered nurse	✓		✓		✓	
	Registered nurse	✓				✓	
	Registered nurse	✓				✓	
	Physician	✓					
	Physician	✓					
**Nephrology outpatient clinic**							
	Head of clinic (physician)			✓		✓	
	Registered nurse	✓		✓		✓	
	Registered nurse	✓					
	Registered nurse	✓					
	Physician	✓					
	Physician	✓					
	Health support personnel	✓					
	Health support personnel					✓	
**Other stakeholders**							
	Patient participant			✓			
	Patient participant			✓			

^a^All stakeholders participated in the development study [41], except for one head of the clinic and one health support personnel.

^b^Participated in data collection.

### Data Collection

#### Focus Groups

HCPs were invited to participate in focus groups, a method suitable for exploring attitudes and experiences, and to encourage group discussion [44]. The HCPs from the nephrology (n=6) and endocrine (n=5) clinics participated in separate groups to explore context-related determinants and key aspects of an implementation plan (Table 1). The focus groups were facilitated by the first (BS) and last (EB) authors. Both focus group sessions lasted approximately 50 minutes and were recorded with a digital voice recorder and transcribed verbatim.

#### Project Steering Committee Meetings

A total of 2 project steering committees were established, one for each participating clinic, to promote leadership and stakeholder engagement in the intervention and to ensure input on the process of tailoring the intervention to fit contextual needs. Participants in each of the 2 project steering committees represented either nephrology (n=3) or endocrine (n=3) outpatient clinics (Table 1). The first (BS) and last (EB) authors facilitated the committee meetings. Each group met twice in the preimplementation phase of the study, which lasted for 1 year. All committee meetings lasted approximately 60 minutes and had a set agenda with topics to discuss (eg, workshop preparation and integration of the intervention into practice). Data collection from committee meetings was based on written minutes made by the last author (EB) during meetings. Each participant received and approved the minutes before the analysis.

#### Workshop

On the basis of the project steering committee meeting discussions and decisions, a joint workshop (n=7) for both participating clinics was considered expedient to share insights and experiences and to identify determinants for tailoring and key aspects for implementation (Table 1). A workshop with HCPs from both clinics was therefore conducted to gain further insight into participants’ reflections and expectations about the *InvolveMe* intervention and elaborate on how to implement the intervention in the 2 clinics [45]. Workshop participants were invited to share their reflections through an exercise in which they were presented with the 5 operationalized domains of CFIR to facilitate narration (Multimedia Appendix 2). The presentation of each domain was followed by group discussions on potential facilitators and barriers. Thereafter, Post-it notes were used to present group reflections and encourage discussions between participants from the 2 clinics. The workshop was facilitated by the first (BS) and last (EB) authors and lasted 180 minutes, including a 15-minute break. Data collection from the workshop resulted in a report based on written notes made by the last author (EB) and pictures of the written Post-it notes made and shared by workshop participants.

### Analysis

Transcripts, meeting minutes, and notes from the 3 data collection activities were deductively analyzed as one data set, based on thematic analysis by Braun and Clarke [46,47], and into the 5 domains of CFIR (ie, themes). The first author (BS) led the analysis process, which involved 2 coauthors (EB and CV). The first step was to read through and become familiar with the transcripts, meeting minutes, and notes. Early impressions were captured during the writing process. Next, the data were coded (ie, coding by CFIR constructs) using an Excel spreadsheet. Quotes from the focus group transcripts were copied and pasted into the spreadsheet along with text sections from meeting minutes and notes. Colors were used to mark data based on sources. The codes were then resorted and re-evaluated based on the CFIR domains and constructs. Through regular coauthor meetings (BS, EB, and CV), codes were discussed and revised to reach a consensus. Codes that did not appear to fit any of the CFIR constructs were also re-evaluated. The analysis was then refined, and the results were written and reviewed. In the final step, quotes were chosen for representation.

### Ethics

The study was performed in accordance with the Helsinki Declaration and approved by the Department for Data Protection and Information Security (equivalent to an institutional review board) at Oslo University Hospital (20178/9223). Written informed consent was obtained from all participants (ie, HCPs and patient representatives). To guarantee confidentiality, the transcripts were coded with project ID numbers and stored on a secure server for sensitive research data, as required by the Department for Data Protection and Information Security at Oslo University Hospital. Only the first author and project administrator (BS) and last author and principal investigator (EB) had access to the code connecting the project ID numbers and the actual participant’s name. Owing to the design and implementation emphasis of the study, the need for study approval was waived by the Regional Committee for Medical and Health Research Ethics for South East Norway, which is in line with the Norwegian legislation [48].

## Results

### Overview

Data were examined and analyzed into 18 CFIR constructs relevant to the study purpose. The constructs are presented by the domains of CFIR, which include description and considerations regarding tailoring of the *InvolveMe* intervention to the intended context (Tables 2-5) and identification of key aspects for the implementation plan (Table 6). A brief description of the relevant CFIR construct is provided to support the interpretation of the results.

**Table 2:** Intervention Characteristics: determinants, tailoring, and identification of key aspects for implementation planning.

Construct and study results	Considerations regarding determinants	Tailoring and key aspects
Intervention Source: intervention considered as internally developed	Facilitator: the involvement in the development study [41] and this study may promote ownership to the intervention, which may support intervention implementation.	Key aspect: ownership
Relative Advantage: the intervention as an advantage to current practice	Facilitator: HCPs^a^ pointed to aspects perceived to be advantages of the intervention. This may be considered as a positive attitude to what is being implemented.	Key aspect: a positive attitude to implement the intervention
Adaptability: use of existing system	Facilitator: participants perceived the previous integration of *InvolveMe* in a patient portal to be beneficial. This may support acceptance and adoption	Key aspect: system acceptance and adoption
Adaptability: a new work task; the secure assessment feature	Barrier: The assessment was a new work task for HCPs, which caused a concern for increased workload and potentially increased time pressure on consultations.	Tailoring: the assessment feature was condensed to a brief list and refinement was made to the summary
Trialability: a need to test before the clinical trial	Barrier: HCPs highlighted that a pilot study would be important to test the intervention and the implementation strategies. A test of the intervention would also inform HCPs that were not formerly involved in the research project and potentially address concerns in advance.	Tailoring: decision, agreed upon by all parties involved, to conduct a pilot study
Complexity: lack of integration between EPR^b^ and patient portal	Barrier: it was recognized as important to improve accessibility and avoid paper printouts of the assessment summary.	Tailoring: the summary was created in a format that could be copied and pasted from the patient portal and into the EPR
Complexity: messages sent directly to the physicians	Barrier: it was considered important to tailor the intervention to suit the physician’s clinical workflow to succeed with intervention implementation.	Tailoring: a shared email inbox with a dedicated triage moderator was established

^a^HCP: health care provider.

^b^EPR: electronic patient record.

**Table 3:** Outer Setting: determinants, tailoring, and identification of key aspects for implementation planning.

Construct and study results	Considerations regarding determinants	Tailoring and key aspects
Patient Needs and Resources: *InvolveMe* could potentially contribute to less anxiety	Facilitator: HCPs^a^ described patients being worried and anxious early in the disease trajectory. *InvolveMe* could be beneficial to patients in terms of increased information and thereby potentially help patients avoid or experience less anxiety.	Tailoring: to provide the intervention as early as possible to patients
Patient Needs and Resources: patient’s motivation to use *InvolveMe*	Facilitator: it can be difficult to reach HCPs on the telephone. *InvolveMe* may have the potential to represent a place where patients can get in contact with HCPs.	Key aspect: motivated patients could contribute to HCPs implementing *InvolveMe*
Patient Needs and Resources: patient acceptance—use of a patient portal	Facilitator: the potential for intervention integration into a patient portal seemed acceptable. This supports findings from the development study [41].	Key aspect: intervention acceptance and adoption
Patient Needs and Resources: patient acceptance—use of a digital health service	Barrier: HCPs described being concerned that *InvolveMe* might be technically demanding for some patients. Therefore, *InvolveMe* was designed to be a voluntary supplement to standard care, not a replacement. The assessment in *InvolveMe* can act as preparation before consultations. To make this clear to all patients, relevant information should be provided.	Tailoring: provide patients with tailored information about the intervention

^a^HCP: health care provider.

**Table 4:** Inner Setting: determinants, tailoring, and identification of key aspects for implementation planning.

Construct and study results	Considerations regarding determinants	Tailoring and key aspects
Structural Characteristics: 2 outpatient clinics organized differently from each other	Facilitator: knowledge about the different organization and staffing may be of importance for tailoring the intervention to fit each outpatient clinics (ie, the moderator functioning).	Tailoring: one clinic designated a nurse to be the moderator, and the other clinic designated health support personnel
Network and Communication: weekly meetings for activity planning	Facilitator: existing meetings were considered appropriate and feasible to discuss and evaluate the implementation process in the research project.	Key aspect: use of existing weekly meetings to monitor implementation process
Culture: interest in innovations	Facilitator: an interest in innovations may provide opportunities to interact with end users (here HCPs^a^) regarding the intervention. This has the potential to support a collaborative relationship between researchers and HCPs.	Key aspect: a collaborative relationship
Tension for Change: improve patient follow-up	Facilitator: HCPs perceived the current situation as demanding, which could contribute to strengthened motivation to change practice (ie, intervention implementation).	Key aspect: monitoring the number of phone calls and the measurement of HRQoL^b^ before and after intervention to visualize change
Leadership Engagement: the heads of the clinics were engaged and active	Facilitator: by their participation in the research project, the heads of the clinics display their commitment and accountability, which may contribute to staff engagement and support a culture for change.	Key aspect: providing anchoring and acceptance for the intervention and a change of practice

^a^HCP: health care provider.

^b^HRQoL: health-related quality of life.

**Table 5:** Characteristics of Individuals: determinants, tailoring, and identification of key aspects for implementation planning.

Construct and study results	Considerations regarding determinants	Tailoring and key aspects
Knowledge and Beliefs: a positive attitude about using a digital intervention such as *InvolveMe*	Facilitator: a positive attitude may act as a facilitator for the implementation process. Reflection on how to maintain a positive attitude throughout the implementation process should be done to establish a close researcher-clinician relationship. The provision of positive feedback along the implementation process might contribute to maintenance of use and collaboration.	Key aspect: maintaining the positive attitude

**Table 6:** Process: determinants and identification of key aspects for implementation planning.

Construct and study results	Considerations regarding determinants	Key aspects
Planning: lack of information and assignment of responsibility may reduce the motivation of HCPs^a^	Barrier: providing information and intervention guidance to staff involved in intervention implementation could include providing project information at meetings and brief updates via email or other information channels to all staff members. Meetings and updates could also allow for information exchange on implementation strategies. Easy access to researchers and technical support in case of questions may be of importance.	Providing: Timely information Someone to call on a specific number Technical training and support Joint project steering committee meetings for mutual exchange of experiences
Engagement: attracting and involving HCPs	Facilitator: some participants initiated writing abstracts to present study details at local and national conferences.	Maintaining engagement
Opinion Leaders	Facilitator: physicians (and head of clinics) were described by some of the nurses as filling an Opinion Leader role.	Involving Opinion Leaders in implementation
Implementation Leaders	Facilitator: participants of the project steering committee were suggested as filling the positions as Implementation Leaders.	Involving members of steering committees in implementation
Champions	Facilitator: registered nurses with a responsibility for the project were seen as potential Champions and drivers of the implementation, inspiring, motivating, and helping other staff members.	Involving registered nurses in implementation
External Change Agents: provide support to clinics	Facilitator: the clinics wanted a designated external facilitator from the research team to provide support for staff members in implementation.	First author (BS) designated as External Change Agent in this study

^a^HCP: health care provider.

### Intervention Characteristics

*Intervention Source* is defined as the key stakeholders’ perception of whether the intervention is externally or internally developed [38]. Most participants were involved in activities to prepare the content and development of the system underlying *InvolveMe*, as described elsewhere [41]. The *InvolveMe* intervention was collaboratively developed within the hospital, and the participating HCPs expressed a perception of *InvolveMe* ownership.

*Relative Advantage* refers to the stakeholder’s perception of the advantage of implementing the intervention rather than an alternative solution [38]. Although symptom assessments were a part of routine consultations, they were performed based on the preference and prioritization of the HCP and the history of the patients. Most participants perceived that the *InvolveMe* intervention could be an advantage compared with current practice where there is no digital communication between patients and HCPs. The participants reported that such a digital intervention could increase patient safety; raise awareness about the patient’s perspective (ie, symptoms and informational needs); and improve patient-provider communication, patient satisfaction, and HRQoL. An intervention that could document contact between patient and provider and reduce the number of phone calls from patients was seen as warranted. One participant stated:

An email is much less disruptive than a phone call. An email I open when I have some spare time, while the phone call I have to answer while in the middle of something, while doing something else.

HCP 10, focus group

*Adaptability* refers to the degree to which an intervention can be adapted, tailored, and refined to meet local needs [38]. The participants shared their opinions on how they thought *InvolveMe* could fit into existing workflows in the clinics. The use of an already existing system was perceived as positive. Participants perceived that there were “already too many digital clinical systems” and that it was beneficial for *InvolveMe* to be integrated into an excising system (ie, patient portal) [41]. Some HCPs expressed concern that the intervention would introduce additional work tasks in an already hectic work environment. These concerns were raised surrounding worries that patients might complete extensive assessments, expecting everything to be addressed in the consultation. One participant stated:

If it becomes one more thing I have to deal with when meeting a patient for half an hour, we’ll have to start considering extending the consultation time.

HCP 6, focus group

*Trialability* relates to the ability to test the intervention on a small scale in the organization and be able to reverse course if warranted [38]. Participants were positive for participating in a clinical trial to test *InvolveMe*. However, HCPs raised concerns about carrying out the planned trial without them being able to test the intervention in advance.

*Complexity* is defined as the perceived difficulty of implementation, reflected by duration, scope, radicalness, disruptiveness, centrality, intricacy, and the number of steps required for implementation [38]. The participants stated that the digital communication tool must be intuitive and easy for them to use. As expressed by one participant:

It has to be something that is intuitive and easy to answer and...something you don’t spend a lot of extra time on.

HCP 9, focus group

Although *InvolveMe* could be integrated into a patient portal, the participants expressed concern that the intervention, because of data protection and privacy regulations, likely would not be allowed to communicate directly with the hospital EPR. If current regulations would require that paper printouts from *InvolveMe* had to be manually scanned into the EPR, rather than received directly from the patient portal, participants were concerned that this would add to their workload. The participating physicians also raised concerns about receiving secure messages directly to an individual mailbox, without some form of triage. There were also some concerns that the message functionality might become more like a chat, with messages going back and forth between patients and HCPs, potentially increasing the time HCPs spend communicating with each other for management of the patients’ many questions.

### Outer Setting

The construct of *Patient Needs and Resources* concerns the extent to which patients’ needs and facilitators and barriers to meeting these needs are accurately known and prioritized by the organization [38]. The participating HCPs explained that they, based on their own experience, perceived patients as the most worried and anxious early in the disease trajectory. They described a structured follow-up for the 2 categories of patients, with room for improvement. As expressed by one participant:

That’s also what we, me too, have been thinking about for many years when it comes to our patients, that they come to the 3-month check-up and they have questions that we could have answered for them [before the time of check-up], but they’ve had no place to pose their questions before consultation.

HCP 2, focus group

HCPs described that they thought most patients would be motivated to use *InvolveMe* and that the intervention could improve patient-provider communication related to symptoms, needs, and preferences, but also serve as a secure digital channel where patients knew that they could get in touch with their HCPs between consultations. This aspect was also discussed in the workshop. One participant described the following:

Satisfied patients, they will feel more seen and heard.

Post-it note, workshop

The use of the existing system was considered positive for patient use. However, the HCPs were concerned about various aspects of patient acceptance. They expressed thoughts that some patients might be afraid of losing in-person contact with their HCPs and that digital communication might not suit all patients. This issue was particularly raised as a digital intervention could potentially require a level of digital competence that some patients might not have. One participant stated:

Some patients might be afraid to use technology.

Post-it note, workshop

Adding to the HCP input, the patient participants in this study supplemented patient input from the development study [41] and strengthened the patient’s voice by providing direct input on the *InvolveMe* intervention. They were very positive toward the use of *InvolveMe* and expressed their view that digital patient-provider communication would strengthen patient follow-up.

### Inner Setting

The construct of *Structural Characteristics* is explained as the social architecture, age, maturity, and size of an organization [38]. The 2 included outpatient clinics were organized differently from each other, although both clinics described staff stability. One clinic was larger than the other and included 2 registered nurses and several physicians. This clinic also had several health support personnel who organized much of the patient-administrative work for registered nurses and physicians. The other clinic included registered nurses and physicians in a relatively small HCP group, which was perceived as an advantage by the HCPs in question in terms of implementation. One participant stated:

It is probably an advantage that we are a small group, and not thousands of people.

HCP 5, focus group

The construct of *Network and Communication* involves the nature and quality of social networks and the nature and quality of formal and informal communication in an organization [38]. Both clinics had weekly meetings for activity planning, where research projects, including this study, were discussed.

*Culture*, as a construct, includes the norms, values, and basic assumptions of a given organization [38]. The HCPs reported that they were generally interested in innovations. This interest was also displayed in attendance and discussions at presentations and meetings about *InvolveMe.* Most of the participating HCPs also pointed to the potential for improved symptom management through interventions such as *InvolveMe*. One participant stated:

It’s the issue of identifying the patient’s problem...that we sometimes struggle to capture.

HCP 4, focus group

*Tension for Change* is the degree to which HCPs perceive the current situation as intolerable or needing change [38]. With regard to digital patient-provider communication, there was a general tension for change among all groups of HCPs in this study. All participants described receiving many phone calls from patients, and that they needed and wanted an easier method for patient follow-up than what current practice allowed, suggesting that digital communication could be one way to improve this issue. One participant stated:

There will be less “noise” if we have one of those electronic communication channels...then we would have the opportunity to convey something, and at the same time reduce the patient’s level of anxiety.

HCP 4, focus group

*Leadership Engagement* refers to the commitment, involvement, and accountability of leaders regarding implementation [38]. The heads of the participating clinics were positive and engaged members of the project steering committee. They were also supportive and involved in the research project, facilitating and participating in research activities and allocating clinic personnel to participate in research project activities and meetings.

### Characteristics of Individuals

The construct of *Knowledge and Beliefs* about the intervention involves individuals’ attitudes and the value placed on the intervention and familiarity with facts, truths, and principles related to the intervention [38]. The participants expressed, for the most part, a positive attitude toward using *InvolveMe* and stated that they believed the use of such an intervention could improve clinical practice through highlighting the importance of good patient-provider communication related to symptom management. One participant stated:

Being able to clarify some expectations makes it easier to...the patient is better prepared for consultation, they understand what they are struggling with and why they come in for consultation...it will potentially make it easier to talk to them when some things are clarified in advance...

HCP 4, focus group

Participating HCPs expressed that digital interventions, such as *InvolveMe,* should be a part of modern practice. The positive attitude toward an intervention such as *InvolveMe* was also expressed in other ways, for example, written on a Post-it note:

Will provide structure to the workday.

Post-it note, workshop

HCPs also stated that they believed that such an intervention could make patients feel safe and cared for. One participant said:

Possibly an increased level of security [for the patient] provided by a communication channel that is not filtered through a switchboard...

HCP 5, focus group

### Process

The construct of *Planning* is explained as the degree to which a scheme or method of behavior, and tasks for implementing an intervention in advance, corresponds with the consideration of the quality of those schemes or methods [38]. The participants in this study stated that a lack of information and assignment of responsibility could potentially reduce HCP motivation. The importance of providing information and guidance for use to everyone involved at the clinics was highlighted:

When switching to new systems, it is always important to have an easily accessible support person who can help solve issues right away.

HCP 5, focus group

The heads of the participating clinics suggested joint project steering committee meetings to exchange information on implementation strategies in the implementation process. In addition, availability from someone from the research team, including the possibility to call if the HCPs had any questions, was suggested. The need for technical support and training was also suggested:

Some training in the use of the software maybe...

Yes, but I often think we get too much of that...

Agreed, but not too long in advance then, as it is so easy to forget. But you could get help with specific things that you wonder about, and then you learn and acquire knowledge, while if you’re sitting in a classroom, learning about a lot of things that you can’t really easily relate to...

Discussion between HCP 6 and 9, focus group

The construct of *Engagement* involves attracting and involving appropriate individuals in the implementation and use of the intervention through a combined strategy of social marketing, education, role modeling, training, or similar activities [38]. There was definite engagement in the planned intervention in this study. For example, some participants initiated writing abstracts to present study details at local and national conferences. This initiation was discussed in project steering committees, and the research team allocated responsibility for the writing process. Abstracts written for the part of the process also received two Best Poster Awards and a Meritorious Abstract Award [49] and contributed to maintaining engagement.

*Opinion Leaders* are individuals in an organization who have formal or informal influence on the attitudes and beliefs of their colleagues regarding the implementation of the intervention [38]. Physicians (and head of clinics) were described by some of the nurses as filling an *Opinion Leader* role. Participants of the project steering committee were suggested to fill the positions as *Implementation Leaders*. Registered nurses who were responsible for the project were seen as potential *Champions* and drivers of the implementation.

*External Change Agents* are individuals who are affiliated with an outside entity and who formally influence and facilitate interventions in a desirable direction [38]. By facilitating the project steering committee meetings, the first (BS) and last (EB) authors potentially influenced and facilitated the intervention as *External Change Agents*. In addition, the participants suggested a facilitator from the research team to be available to the clinic staff members for support during the implementation process.

## Discussion

### Principal Findings

This study identifies the determinants using the CFIR framework [38] to inform tailoring of the *InvolveMe* intervention and to identify key aspects for implementation planning based on context.

The identification of determinants in this study supports findings from existing literature [23,33,50]. However, the influence of context on implementation outcomes must be considered to understand the need to tailor interventions [51]. To the best of the authors’ knowledge, descriptions of how identified determinants can be used to tailor interventions to context are largely lacking. The HCPs participating in this study were mostly positive toward implementing the digital communication intervention *InvolveMe* and perceived the intervention as having the potential to improve patient-provider communication. This is in line with existing research showing that improvements in communication can act as facilitators in eHealth implementation [24,50].

In the development study [41] preceding this study, the participants voiced a concern about lack of integration with existing systems, which corresponds with findings from other studies where lack of accessibility and fit into organizational structures have been identified as barriers to implementation of eHealth interventions [23,33,50]. Therefore, the *InvolveMe* intervention was integrated into a patient portal already in use by patients and HCPs. What was initially perceived as a barrier in the development study [41] was hence turned into something, perceived by participants, beneficial in this study. This confirmed the decision made in the development study [41], acting as a potential facilitator for intervention implementation and potentially improving acceptance and adoption [33].

The HCPs in this study voiced some concerns regarding the assessment feature in terms of being a new work task that could potentially increase the workload. The assessment feature was therefore condensed to a brief list and refinements were made to the assessment summary by conducting several user tests in close collaboration with HCPs, patient participants, and the research team. This strategy is supported by the literature, showing user-friendliness and integration into care as known facilitators for the implementation of eHealth interventions [22,50,52]. In addition, this strategy can prevent the need for intervention redesign, which is likely to delay use and increase costs, which are known barriers to eHealth interventions [33].

The involvement of relevant stakeholders is a known facilitator of implementation [50,53]. Although stakeholders were involved in the development [41] of the *InvolveMe* intervention, the participants in this study provided valuable additional information for the tailoring of the intervention, including the need to test the intervention before any upcoming clinical trial. Participants in this study raised concerns about potential implications for clinical workflow and workload when using a digital communication intervention. Concerns about increased workload are a well-known barrier to the implementation of eHealth interventions [28,50]. A growing point of importance is also to tailor eHealth interventions to existing clinical workflows, minimizing potential burdens [30]. A moderator function for triaging secure messages should therefore be organized in a flexible way, depending on the clinic organization and available HCPs. Such tailoring to the local context may facilitate intervention integration into clinical workflow, a known facilitator for implementation of eHealth interventions in practice [22,50].

In this study, HCPs also expressed concerns about patient acceptance of a digital communication intervention, described as a lack of digital competence among some patients. This concern is supported by patient education literature [20,24,54]. However, there are some indications that it is feasible to deliver eHealth interventions to improve eHealth and health literacy skills among patients with chronic health conditions [22,54]. To be able to offer interventions, such as *InvolveMe* to patients, regardless of digital competence, studies have suggested employing blended care models, involving a mixture of in-person, technology, or telephone contact as a way to help facilitate use [23,55,56]. Furthermore, alternating health care delivery between digital communication and in-person meetings has been described as a way to avoid losing in-person contact with patients [23].

To ensure successful implementation of an eHealth intervention in a certain context, the need to develop and follow an implementation plan is widely recognized [36] and the lack of such a plan is considered a barrier to implementation [33]. In this study, results from the CFIR *Planning* construct provided insight into the participants’ thoughts on how to involve key stakeholders, secure leadership support, and how to provide information training and coaching. These factors have previously been described as important for incorporation into an implementation plan [32,37]. In addition, several facilitators (eg, ownership, positive attitude, and system acceptance) identified in the other CFIR domains were considered important to build on (ie, leverage facilitators) when planning for implementation. HCPs struggling with the use of technology are a known barrier in the implementation of eHealth interventions [23]. Training of HCPs is therefore a preferred and widely used implementation strategy [53], and a combination of software training and training in how to incorporate the intervention into daily clinical workflow may be required [30,31]. In this study, it was hence considered an important aspect to include training and follow-up of HCPs in the implementation plan. Training of designated clinicians who would subsequently train others to use *InvolveMe* was planned [32]. External support (ie, provided by a member of the research team) was also identified as a key aspect of the implementation plan.

The heads of both clinics participating in this study were involved in the development study [41] and in the tailoring and implementation planning. Several studies have shown that implementation strategies that encourage leadership support and engagement are crucial to implementation success [30]. Leaders are often seen as providers of new knowledge and as key influencers related to implementation initiatives, including facilitating effective teamwork and cultivating a culture of learning [57]. In addition, leaders can assign dedicated staff to perform the required change, which may ease workload and the concern for increased work [57]. Therefore, strategies targeting leaders, such as continuing the project steering committee meetings, should be considered key aspects to include in the implementation plan of interventions, such as *InvolveMe,* into outpatient clinics. In addition, carrying out implementation preparation workshops (Multimedia Appendix 2), as in this study, might also capture local knowledge of what works and not, knowledge that can be shared between implementation sites [32].

In this study, the project steering committee meetings were also intended to build a coalition between patient participants, HCPs, and the research team to cultivate a good relationship in the implementation effort, another described implementation strategy [32,37,53], and thus a key aspect to include in an implementation plan. Collaborative relationships are crucial for implementing plans and, through a social exchange communicating the potential impact of innovations, for the implementation of interventions, such as *InvolveMe*, into clinical practice may be facilitated [57].

### Strengths and Limitations

This study has some limitations that need to be addressed. *First,* the study was conducted at a single university hospital. This might limit transferability to other settings. However, the inclusion of 2 outpatient clinics, following up 2 different patient categories, might increase transferability to other settings. *Second*, the study had a relatively small sample size, a factor potentially limiting transferability. A small sample size may limit the ability of data to describe the entire local context at the clinics involved. However, the study was performed in outpatient clinics where the implementation of the intervention is planned to take place, thus enabling identification of local determinants and key aspects that may be crucial for an implementation plan. *Third,* there was an imbalance in the number of HCPs compared with patients in this study. Traditionally, implementation involves the improvement of HCP practices [35]. CFIR does not differ from this tradition, as CFIR places patients under the *Outer Setting* domain, where only one single construct is intended to capture patients’ needs and resources [38]. As such, patients are considered to have a peripheral role in their implementation. However, this study examined all perspectives and interplay of all stakeholders involved, including patient participants, which helped to illuminate stakeholder aspects and may help increase the likelihood of successful implementation to practice [33]. In addition, even with a limited number of patient participants, patient participants’ experience from the development study [41] and subsequent direct input on various topics in this study may have ensured relevance and reliability from the patient perspective. *Fourth,* the perceived facilitators and barriers of participants in this study might not necessarily correspond to facilitators and barriers experienced in clinical practice in general. However, recent evidence indicates that the limited use of tailoring to context could explain the limited implementation success [30]. Knowledge generated during preparation for implementation can contribute to intervention tailoring and context-specific individualization, which implies that stakeholders’ needs are more likely met, and hence intervention design and implementation preparation are improved [58].

This study has some strengths. Applying a structured and comprehensive framework such as CFIR within the field of implementation is considered to be a strength guiding data collection and analysis [59]. Identifying and describing determinants that affect implementation, as well as identifying key aspects for implementation planning, are also strengths [59]. Furthermore, the use of CFIR in this study provided a common language through the use of constructs and definitions for the analysis of data and thus may provide comparable results that may make it easier to assess why and how certain elements work. However, it should be noted that the strength of CFIR as a comprehensive framework may also be a weakness. CFIR does not distinguish between the relative importance of all of its constructs, which may imply that the details necessary for implementation success could be lost if trying to capture as many constructs as possible. In this study, a number of CFIR constructs were not covered by the data collection, as only discrete but significant themes and questions were targeted (Multimedia Appendices 1 and 2). Another challenge using CFIR is that some constructs are broad and difficult to capture and some may overlap with other constructs. Further descriptions and explanations related to constructs could enhance the CFIR and thus make the framework more intuitive to use when planning for implementation.

### Future Directions

The ongoing COVID-19 pandemic has triggered a significant need for a wide range of digital communication services between patients and HCPs and has led to increased demand for digital intervention from within the health care services themselves. As such, and with the tremendous challenges posed by this significant health challenge, the pandemic might turn out to be a powerful facilitator for the implementation of digital interventions in health care services.

This study revealed some specific aspects that need to be investigated in future research. In particular, the results show that a pilot study may contribute to identify gaps and inform further necessary tailoring of the intervention and an implementation plan (ie, strategies) before clinical trials. This emphasizes that, regardless of stakeholder involvement in intervention development, a pilot test should always be considered.

Future studies should also aim to better understand how the CFIR framework can inform an implementation planning process in terms of tailoring interventions before implementation and the selection of implementation strategies based on identified determinants. In addition, refinements of the CFIR to strengthen the patient-related constructs and make the framework easier to apply would be beneficial for researchers and for HCPs conducting implementation in clinical practice.

### Conclusions

This study contributes to the field of implementation science by using identified determinants to inform the tailoring of a digital communication intervention (ie, *InvolveMe*) and to identify key aspects of an implementation plan to context. Important intervention tailoring aspects identified were adjustments to the digital features and adjustments to fit the clinical workflow as well as recommendations to conduct a future pilot study before testing in larger clinical trials. Future research into the implementation of digital communication interventions should focus on the early identification of determinants and attention to tailoring to address barriers and leverage facilitators. In addition, key aspects of implementation planning should be identified, raising the probability of implementation success.

## Appendix

Multimedia Appendix 1 Focus group guide with Consolidated Framework for Implementation Research domains.

Multimedia Appendix 2 Workshop theme guide with Consolidated Framework for Implementation Research domains.

## Abbreviations

CFIR: Consolidated Framework for Implementation Research
EPR: electronic patient record
HCP: health care provider
HRQoL: health-related quality of life
