# Supplementary material for: A Digital Patient-Provider Communication Intervention (InvolveMe): Qualitative Study on the Implementation Preparation Based on Identified Facilitators and Barriers
Source: J Med Internet Res. 2021 Apr 8;23(4):e22399. doi: 10.2196/22399 (PMC8294341; doi:10.2196/22399)
Supplement: Multimedia Appendix 1 [file jmir_v23i4e22399_app1.pdf]

# InvolveMe
# Focus Group Guide
## Health care providers

**Theme: Implementation of a digital patient- provider communication intervention**

**Introduction by first-author (BS):** In this project we have developed a digital intervention to be used by you and your patients. The intervention provides a secure symptom and needs assessment to be used prior to hospital visits as a preparation for consultations for patients and health care providers and the opportunity for follow-up before, after and in between hospital consultations - with an option for secure message (email) between patients and health care providers. We need your thoughts and suggestions on this aim in order to make the intervention suit your daily workflow.

**What do you think would be potential advantages that the digital intervention may have for:**

- The patient group [Intervention Characteristics, Outer Setting]
- Health care providers and managers [Intervention Characteristics, Inner Setting]
- The way you work (The work process) [Intervention Characteristics, Inner Setting]
- The outpatient clinic [Intervention Characteristics, Inner Setting]

**Think back on the recent implementation of the new version of the electronic patient record (EPR)* at the outpatient clinic**

- What challenges did the implementation of the EPR face? [Inner Setting]
- What challenges can implementation of the digital tool face? [Inner Setting]
- Do you have suggestions for how challenges can be meet? [Process]

**Was there anything that worked well in relation to the implementation of the EPR?**

- If so, what was it? [Inner Setting, Process]
- What contributed to its success? [Process]

**What does it take to succeed with implementation of the digital tool?**

- What can you do to make it work? [Process]

**In conclusion:** Is there anything that would be particularly important for us to keep in mind?

*We had made an inquiry an knew beforehand that this was a resent implementation conducted at the outpatient clinics.
