# Supplementary material for: A Digital Patient-Provider Communication Intervention (InvolveMe): Qualitative Study on the Implementation Preparation Based on Identified Facilitators and Barriers
Source: J Med Internet Res. 2021 Apr 8;23(4):e22399. doi: 10.2196/22399 (PMC8294341; doi:10.2196/22399)
Supplement: Multimedia Appendix 2 [file jmir_v23i4e22399_app2.pdf]

InvolveMe
Workshop Theme Guide
Health care providers

Theme: Implementation of a digital patient- provider communication intervention

Introduction by first-author (BS): The aim of this workshop is to gain knowledge about potential facilitators and barriers to the use of a digital patient -provider communication intervention, and to use this knowledge to tailor the intervention and prepare for implementation.

Theme: Desired use of the *InvolveMe* intervention (facilitators & barriers)**.**
Keywords to help start the group discussion:

- Who should be responsible for what? [Intervention Characteristics]
- What obstacles can arise? [Intervention Characteristics]
- Potential advantages and disadvantages? [Intervention Characteristics]
- Any risks? [Inner Setting]

Theme: The patients who will be offered the *InvolveMe* intervention (facilitators & barriers for patients use).
Keywords to help start the group discussion:

- What is important for patients who will be offered the intervention? [Outer Setting]
- Patients' needs [Outer Setting]
- Patients motivation for use? [Outer Setting]
- Is the tool suitable for every patient? [Outer Setting]
- Potential advantages and disadvantages? [Intervention Characteristics, Outer Setting]

Theme: **The outpatient clinics where the patients are treated** (facilitators & barriers)
Keywords to help start the group discussion:

- Workflow [Inner Setting]
- Disruption of the of the workflow [Inner Setting]
- Collective responsibility? [Inner Setting]
- Interdisciplinary collaboration? [Inner Setting]
- Potential advantages and disadvantages? [Inner Setting]
- Any risks? [Inner Setting]

Theme: Health care providers at the outpatient clinic (facilitators & barriers).
Keywords to help start the group discussion:

Related to *InvolveMe*:

- Enthusiasm in the outpatient clinic [Characteristics of individuals]
- What about expectations? [Characteristics of individuals]
- What do other staff members at the outpatient clinic say? [Characteristics of individuals]
- Why do you want to do this? [Characteristics of individuals]
- What do you want to achieve? [Characteristics of individuals]
- What is important to HCPs? [Characteristics of individuals]

Theme: Preparations for the implementation of the *InvolveMe* intervention.
Keywords to help start the group discussion:

- The need for training? [Process]
- The need for support? [Process]
- Implementation monitoring? [Process]
- Evaluation of implementation process. [Process]
- Organization of management related to the implementation? [Process]
